# Monensin ameliorates cadmium-induced hepatic injury in mice, subjected to subacute cadmium intoxication

**DOI:** 10.1080/13102818.2014.901673

**Published:** 2014-05-09

**Authors:** Juliana Ivanova, Yordanka Gluhcheva, Kalina Kamenova, Sonja Arpadjan, Mariana Mitewa

**Affiliations:** ^a^ Department of Chemistry and Biochemistry, Physiology and Pathophysiology, Sofia University ‘St. Kliment Ohridski’,Faculty of Medicine, Sofia, Bulgaria; ^b^ Bulgarian Academy of Sciences, Institute of Experimental Morphology, Pathology and Anthropology with Museum, Sofia, Bulgaria; ^c^ Department of Analytical Chemistry, Sofia University ‘St. Kliment Ohridski’,Faculty of Chemistry and Pharmacy, Sofia, Bulgaria

**Keywords:** Cd intoxication, ICR mouse model, monensin, chelating agents

## Abstract

This study was designed to evaluate the potential application of monensin as an oral drug for the treatment of cadmium-induced hepatic dysfunction. The study was performed using ICR mouse model. Twenty-seven adult ICR male mice were divided into three groups of nine animals each: control (received distilled water and food ad libitum for 28 days); Cd-intoxicated (treated orally with 20 mg/kg b.w. Cd(II) acetate from the 1st to the 14th day of the experimental protocol); and monensin treated group (intoxicated with Cd(II) acetate as described for the Cd-intoxicated group followed by an oral treatment with 16 mg/kg b.w. tetraethylammonium salt of monensic acid for two weeks). The obtained results demonstrated that the treatment of Cd-intoxicated animals with monensin restored the liver weight/body weight index to normal values, decreased the concentration of the toxic metal ion by 50% compared to the Cd-treated controls, and recovered the homeostasis of Cu and Zn. Monensin reduced the activity of aspartate aminotransferase, alanine aminotrasnferase and alkaline phosphatase in the plasma of Cd-treated animals to the normal control levels and ameliorated the Cd-induced inflammation in the liver. Taken together, these data demonstrated that monensin could be an effective chelating agent for the treatment of Cd-induced hepatotoxicity.

## Introduction

Cadmium (Cd) is a toxic element emitted in the environment during the production of pigments, alloys, Cd-nickel batteries and fertilizers.[1] The main sources of environmental exposure of humans to cadmium are cigarette smoking and food.[1] It has been estimated that the average daily Cd intake from people living in unpolluted regions in Europe lies in the range of 8–30 μg.[2] However, the dose above which Cd causes early health effects is largely unknown.[3] Due to its very long biological half-life,[4] Cd accumulates in the organs causing anaemia,[5] osteoporosis,[6] renal and cardiovascular dysfunctions,[7–9] hepatotoxicity,[10,11] cancer [12] and infertility.[13] The kidneys and liver are the most sensitive organs in oral chronic and acute Cd intoxications.[14–16]

**Figure 1.**
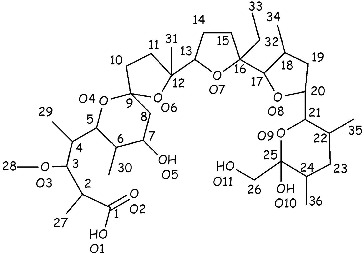
Structure and numbering sequence of monensic acid.

Different antioxidants and chelators have been tested as antagonists of Cd, but the information for their effect on different organs and systems is still sparse.[15,17–23] DMSA (2,3 dimmercaptosuccinic acid) is the conventional antidote applied for the treatment of intoxication with toxic elements.[9,20] It has been reported that the polyether ionophorous antibiotic monensin significantly increases the effectiveness of DMSA for the treatment of lead (Pb) intoxication in rats.[24,25] Monensin (Figure 1) has been used both in poultry for the treatment of coccidiosis and as a growth promoter feed additive.[26] It has been demonstrated that this antibiotic exerts a pronounced cytotoxicity against prostate,[27] colon,[28] myeloma [29] and leukaemia [30] cancer cells, suggesting that it could be applied as an antitumor agent in human medicine. Our recent study [14] proved that monensin, applied as tetraethylammonium salt, significantly reduces the concentration of Cd in the organs of mice subjected to Cd intoxication. Furthermore, we have presented experimental evidence that monensin ameliorates Cd-induced iron (Fe) deficiency and improves haemoglobin, red blood cell distribution width and whole blood viscosity in Cd-treated mice.[31]

These results demonstrate that monensin might be a promising chelating agent for the treatment of Cd-intoxication and lay the ground for studies on the effect of monensin on Cd-induced organ toxicity in animal models. To the best of our knowledge, there is no information regarding the impact of monensin on Cd-induced hepatotoxicity. Hence, the aim of this study was to investigate the effect of monensin on Cd-induced hepatic dysfunction in mice subjected to subacute Cd intoxication.

## Materials and methods

### Animal treatment

Sixty-day-old adult male ICR mice, weighting 25–30 g were obtained from the animal care unit, Slivnica (Bulgaria). The animals were housed at the Institute of Experimental Morphology, Pathology and Anthropology with Museum, Bulgarian Academy of Sciences (BAS) and were maintained at 23 °C under controlled light/dark cycle (12:12 h) in individual, standard hard-bottomed polypropylene cages. The animals had free access to standard diet and were allowed to acclimate for one week prior to dosing. Twenty-seven mice were classified into three groups with nine mice in each group as follows.

The normal control group was given standard diet and had free access to distilled water during the experimental protocol. The second group of animals (Cd-treated controls) received orally Cd(CH_3_COO)_2_ × 2H_2_O (20 mg/kg body weight) once daily for two weeks. The compound was dissolved and given in drinking (distilled) water. During the next 14 days of the experiment, the animals from this group were allowed to drink distilled water and food *ad libitum*.

The third group (monensin-treated mice) was subjected to intoxication with Cd(CH_3_COO)_2_ × 2H_2_O as described above. The Cd-intoxicated animals were treated orally with 16 mg/kg body weight of tetraethylammonium salt of monensic acid (dissolved in the drinking [distilled] water) from day 15 to day 28 of the experimental protocol.

The doses of both compounds, Cd(II) acetate and tetraethylammonium salt of monensic acid, were chosen to be 10% of the corresponding LD_50_ values (Ivanova et al., unpublished results).

On the day 29 of the experimental protocol, all the animals were sacrificed under light ether anaesthesia. The liver of each animal was excised and stored at −20°C prior to analysis. Blood samples were collected in heparinized tubes and centrifuged, and the resulting plasma samples stored at −20 °C. The animal studies were approved by the Ethics Committee of the Institute of the Experimental Morphology, Pathology and Anthropology with Museum, BAS.

### Chemicals

The sodium salt of monensin was obtained from Biovet Ltd. (Peshtera, Bulgaria). Tetraethylammonium hydroxide (Et_4_NOH), nitric acid (HNO_3_) and diethyl ether (Et_2_O) were purchased from Merck (Darmstadt, Germany).

### Preparation of monensic acid

Monensic acid A monohydrate (Figure 1) was prepared from sodium monensin (711 mg, 1 mmol), using the procedure previously reported (589 mg, 85% yield).[32,33] Anal. Calcd. for C_36_H_64_O_12_ (%): H, 9.36; C, 62.77; O, 27.87. Found: H, 8.97; C, 62.95; O, 27.60. Details of the ^1^H and ^13^C NMR spectra of the compound were given previously.[33]

### Assay of aspartate aminotransferase (AST), alanine aminotrasnferase (ALT) and alkaline phosphatase (ALP) in the plasma of the experimental animals

The biochemical analysis of plasma for the determination of the levels of AST, ALT and ALP was conducted in Clinical Laboratory Ramus (Sofia, Bulgaria) using standard protocols for clinical analysis. Laboratory Ramus is certified by the Ministry of Health (Sofia, Bulgaria) to perform biochemical analysis of clinical samples.

### Assay of Cd, copper (Cu) and zinc (Zn) contents in the liver of the experimental animals

The liver was digested with concentrated HNO_3_ (free of metal ions) for 24 h. After evaporation of the samples on a sand bath to approximately 2 mL, 1.5 mL of concentrated HClO_4_ (free of metal ions) was added. The samples were then concentrated to approximately 1 mL and quantitatively transferred to polyethylene centrifuge tubes (15 mL) with distilled water. Blanks were prepared in duplicate. For the determination of the Cd, Cu and Zn content in the liver, a Perkin Elmer AAAnalyst 400 flame atomic absorption spectrometer was used with air-acetylene flame and hollow cathode lamps for Cd (228.8 nm), Cu (324.8 nm) and Zn (213.9 nm). The instrumental parameters were optimized to obtain maximum signal-to-noise ratio for standard analyte solutions aspirated into the flame. The limits of detection for Cd, Cu and Zn were 0.1 mg/L, 0.2 mg/L and 0.1 mg/L, respectively.

The accuracy of the data was checked by analysis of Certified Reference Materials from the International Atomic Energy Agency (IAEA-H-8 (kidney) and IAEA-H-4 (animal muscle). Recoveries from the analysis of the certified reference materials were in the range of 93% to 102%.

### Histopathological analysis

Livers from control and treated mice were fixed in Bouin fixative for 24 h and paraffin-embedded. Paraffin sample sections with 5 μm thickness were stained with hematoxylin and eosin and observed on a light microscope Leica.

### Statistical calculations

The results for the three studied groups are presented as the mean value ± SD (number of studied animals in one group, *n* = 9). To estimate the significance of the differences between the experimental results of two groups, student's *t*-test was applied. The results between two groups were considered significantly different at *p* < 0.05.

## Results and discussion

The hepatotoxicity of Cd has been discussed as a biphasic process.[10] The first stage is determined by the direct action of the metal ion and ischemia due to endothelial cell damage. The second stage is attributed to inflammation as a result of Kupffer cell activation and oxidative stress.[10] The ability of Cd to cause oxidative stress is related with its effect on iron (Fe) homeostasis. Cd replaces the Fe in ferritin and transferrin, thus increasing the concentration of free metal ion and the reactive oxygen species (ROS) formed in the Fenton type reaction.[34,35] In our previous study,[14] we have reported that the polyether ionophorous antibiotic monensin restores the normal values of Fe in Cd-treated mice, suggesting that the treatment of Cd-intoxicated animals with this chelating agent would inhibit the Cd-induced toxicity.

The aim of the present study was to evaluate the potential application of monensin as an oral drug for the treatment of Cd-induced hepatic dysfunction.

**Figure 2.**
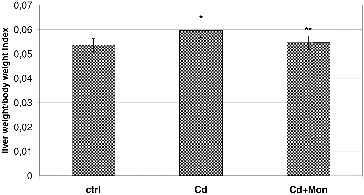
Effect of Cd and tetraethylammonium salt of monensic acid on the liver weight/body weight index in mice subjected to subacute Cd intoxication. Ctrl: normal control mice; Cd: Cd-treated mice; Cd + Mon: Cd-intoxicated mice treated with tetraethylammonium salt of monensic acid. Mean ± SD, *n* = 9; significant difference (*p* < 0.05) between the Cd-treated group and the control (*), and between the monensin-treated group and Cd-intoxicated group (**).

Figure 2 depicts the effect of Cd and monensin on the liver weight/body weight index. The results demonstrate that Cd caused a significant increase of the liver weight, compared to the normal controls (*p* < 0.05). Our finding is in agreement with the studies of other authors,[36,37] where higher liver weight was observed in Cd-treated animals compared to the normal controls. The results reported by Sinha et al.,[38] however, illustrated that Cd decreases the liver weight in Cd-treated animals. The discrepancy of the experimental results obtained by different authors about the effect of Cd on the liver weight is consistent with the conclusion reported by Casalino et al. [39] that the toxicity of Cd, both in experimental animals and humans, is affected by the duration of exposure, the age of the experimental animals, the route of administration, the dosage and the chemical form of the metal. The increase of the liver weight by Cd is most likely a result of Cd-induced inflammation of the hepatic tissue and intracellular vacuolation.[36] The Cd-induced decrease of liver weight is characteristic for severe Cd-intoxications, resulting in a centrilobular necrosis.[38] The treatment of the Cd-intoxicated mice with monensin led to a significant decrease of the liver weight to normal values (*p* < 0.05, Figure 2), demonstrating the ability of the antibiotic to recover Cd-induced alterations in the hepatic function.

**Figure 3.**
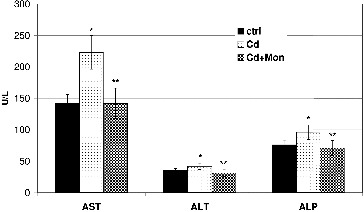
Effect of Cd and tetraethylammonium salt of monensic acid on the levels of AST, ALT and ALP in the plasma of experimental mice subjected to subacute Cd intoxication. Ctrl: normal control mice; Cd: Cd-treated mice; Cd + Mon: Cd-intoxicated mice treated with tetraethylammonium salt of monensic acid. Mean ± SD, *n* = 9; significant difference (*p* < 0.05) between the Cd-treated group and the control (*), and between the monensin-treated group and Cd-intoxicated group (**).

The influence of Cd and monensin on the levels of enzymes: AST, ALT and ALP in the plasma of the Cd-treated mice is illustrated in Figure 3. The results demonstrated that the intoxication of the animals with Cd caused a significant increase (*p* < 0.05) of the three enzymes in the plasma. The AST was most affected by Cd, compared to the ALT and ALP. The enzymes AST and ALT have been used as a quantitative marker for assessing the extent and type of the liver damage.[4,15,36] An increase in levels of these enzymes in the plasma of Cd-treated animals, compared to the normal control, is also reported by Hwang and Wang,[40] Pari and Murugavel,[41] Renugadevi and Prabu, and [42] Pari and Shagirtha [36] and has been associated with the leakage of the AST and ALT from the liver cytosol into the blood stream. ALP has been also related to the status and function of the liver.[36] The treatment of the Cd-intoxicated animals with monensin reduced the levels of the three enzymes in the plasma to normal values.

**Figure 4.**
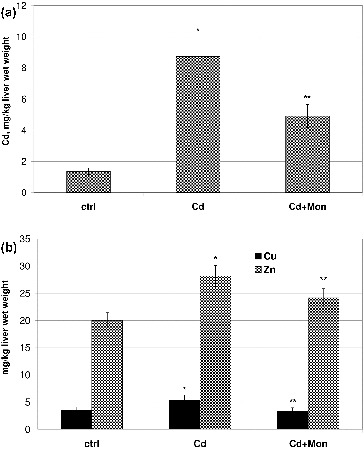
Effect of Cd and tetraethylammonium salt of monensic acid on the concentration of Cd (A) and the concentration of Cu and Zn (B) in the livers of experimental mice subjected to subacute Cd intoxication. Ctrl: normal control mice; Cd: Cd-treated mice; Cd + Mon: Cd-intoxicated mice treated with tetraethylammonium salt of monensic acid. Mean ± SD, *n* = 9; significant difference (*p* < 0.05) between the Cd-treated group and the control (*), and between the monensin-treated group and Cd-intoxicated group (**).

The results from the atomic absorption analysis of the livers of the animals for Cd, Cu and Zn determinations are presented in Figure 4. The intoxication of the animals with Cd resulted in a significant accumulation (*p* < 0.05) of the toxic metal ion in the liver compared to the normal control (Figure 4(A)). Cadmium accumulation in the liver has been considered as a primary mechanism of hepatic damage induced by this metal.[36,43] The treatment of the Cd-intoxicated animals with monensin significantly decreased the Cd concentration in the liver, which demonstrated that monensin binds Cd and promotes its excretion from that organ.

Cd significantly increased by 35% (*p* < 0.05) the concentration of Cu and by 30% the concentration of Zn ions in the liver of the animals compared to the normal controls (Figure 4(B)). The increase of the concentrations of Cu and Zn in the liver of Cd-treated animals has been associated with *de novo* synthesis of metallothionein induced by Cd administration.[44] The studies by Nakagawa et al.,[44] however, demonstrated that another cause for Cd-induced increase of Zn concentration in the liver of Cd-intoxicated animals could not be ruled out. Cd replaces Cu and Zn from the cofactor sites in many metaloenzymes, which could lead to an increase in the concentration of the free metal ions in the liver.[10,38] The treatment of the animals with lower doses of Cd(II) acetate did not affect the homeostasis of Cu and Zn (data not shown).

The treatment of the animals with monensin decreased by 50% the concentration of the toxic metal ion in the liver, restored the level of Cu and significantly reduced the concentration of Zn. The ability of monensin to reduce cadmium concentration in the organs of Cd-intoxicated animals and to recover the homeostasis of the essential metal ions (Cu, Zn, Fe) could be one of the advantages of this chelating agent over other compounds applied in the treatment of Cd intoxications.

**Figure 5.**
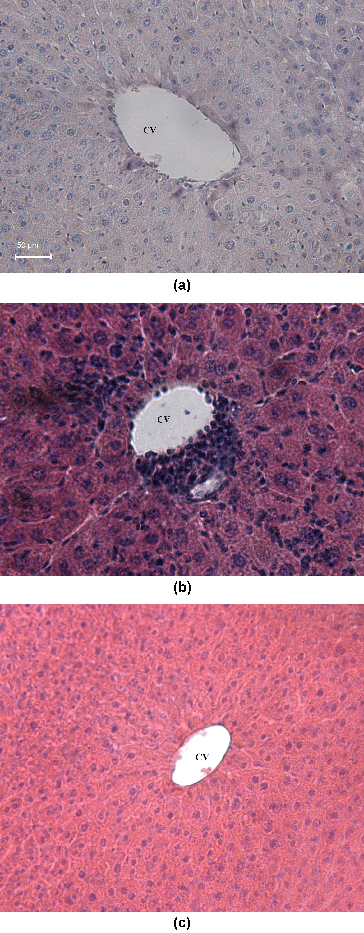
Hematoxylin—eosin stained liver section: normal control mice (A) with normal hepatic architecture around the central vein (CV), 200×; mice treated with Cd(II) acetate (B) with severe leukocyte infiltrations (arrow) in the central vein and distortion of the normal radiating pattern of the hepatocytes, 100×; and Cd-intoxicated animals subjected to therapy with monensin (C), 100×.

The morphological Cd-induced alterations in the liver depend strongly on the dose of the toxic metal ion, duration of the exposure and route of the administration of the metal salt.[10,23,37,45,46] As seen from the results presented in Figure 5(B), the treatment of the mice with Cd(II) acetate induced severe leukocyte infiltrations in the central vein. A distortion of the normal radiating pattern of the hepatic tissue was also observed. Our finding is in good agreement with the studies by Pari and Shagirtha,[36] Gong et al., and [45] and Sk et al.,[37] where Cd-induced alterations of the normal architecture of the hepatic parenchyma with inflammatory cell infiltration is documented. Although some leukocyte infiltrations could be still observed in the liver of monensin-treated mice, the treatment of the Cd-intoxicated animals with monensin significantly reduced the leukocyte infiltrations in the central vein (Figure 5(C)), compared to the Cd-treated controls (Figure 5(B)).

## Conclusions

The results presented herein confirm the ability of monensin to mobilize Cd from the liver of Cd-treated animals and for the first time demonstrate that monensin inhibits Cd-induced hepatic injury. Furthermore, the chelating agent significantly improves Cu and Zn homeostasis in the liver of Cd-treated animals, which could be an advantage of monensin over other chelators tested for treatment of Cd-intoxication. This study expands the current knowledge regarding the biological properties of monensin and proves that polyether ionophores could be promising chelating agents for the treatment of heavy metal intoxication.
